# Epidemiology of distal radius fractures in children and adults during the COVID-19 pandemic – a two-center study

**DOI:** 10.1186/s12891-021-04128-5

**Published:** 2021-03-26

**Authors:** Jarosław Olech, Mariusz Ciszewski, Piotr Morasiewicz

**Affiliations:** 1 Provincial Specialist Hospital in Legnica, Orthopedic Surgery Department, Iwaszkiewicza 5, 59-220 Legnica, Poland; 2 Department of Orthopaedic and Trauma Surgery, University Hospital in Opole, Institute of Medical Sciences, University of Opole, al. Witosa 26, 45-401 Opole, Poland

**Keywords:** Distal radius, Fracture, Covid-19, Epidemiology, Lock-down, Pandemic, Sars-cov-2

## Abstract

**Background:**

Distal radius fractures (DRFs) constitute 15–21% of all fractures**.** There are no detailed data on the possible changes in the epidemiology and treatment of DRFs in children and adults during the Covid pandemic. The purpose of our study was a comprehensive assessment of the impact of the COVID-19 pandemic on distal radius fractures (DRF) epidemiology, including both children and adults and various fracture fixation methods in two large trauma centers in Poland.

**Methods:**

This study compared the medical data on the treatment of distal radius fractures in Poland in two periods: the period of the COVID-19 pandemic (from March 15 to October 15, 2020) and the corresponding period prior to the pandemic (from March 15 to October 15, 2019). We assessed detailed data from two trauma centers for pediatric and adult patients. Outpatients seeking medical attention at emergency departments and inpatients undergoing surgery at trauma-orthopedic wards were evaluated. We compared epidemiological data, demographic data, treatment type, and hospital stay duration.

**Results:**

The total number of patients hospitalized due to DRF during the pandemic was 180, it was 15.1% lower than that from the pre-COVID-19 pandemic period (212). In the case of adult patients, the total number of those hospitalized during the pandemic decreased significantly (by 22%) from 132 to 103 patients. Analysis of the individual treatment methods revealed that the number of adults who underwent conservative treatment was considerably (by 30.3%) significantly lower in the period of the COVID-19 pandemic, from 119 to 83 patients. Compared to 13 patients from the pre-pandemic period, the number of surgically treated adults statistically increased to 20 patients (by 53.8%). Our analyses showed hospitalizations of surgically treated adults to be shorter by 12.7% during the pandemic, with the corresponding hospitalizations of surgically treated pediatric patients to be shorter by11.5%.

**Conclusions:**

Our study showed a significant impact of the COVID-19 pandemic on the epidemiology and treatment of DRFs in children and adults. We found decreased numbers of pediatric and adult patients with DRFs during the COVID-19 pandemic. The pandemic caused an increase in the number of children and significantly increase adults undergoing surgical treatment for DRFs, a decrease in mean patient age, shorter significantly *length* of hospital stay, and an increased number of men with DRFs.

## Background

Distal radius fractures (DRFs) constitute 15–21% of all fractures and are the third most common location of osteoporosis-related fractures [1–14]. The estimated risk of DRF is 9–139/10,000 people per year [3, 4, 8, 11, 12]. There are different DRF fixation methods used in children and adults [2, 6, 7, 9–11].

The COVID-19 pandemic altered the healthcare in the whole world in the year 2020 [13–26]. Although the causative coronavirus (SARS-CoV-2) can infect both adults and children, the majority of children have mild or asymptomatic course of the disease [13]. The COVID-19 pandemic has impeded general access to specialist care and altered the daily clinical practice and admission routines (in both emergency and primary-care settings) [14–17, 19, 21, 22]. Large group of Hospitals changed its working routines to special – crisis mode, cancelling or limitting planned admissions [16, 17, 22, 26]. Some physicians switched to contracted COVID-19, some shortened their office hours to limit the risk of infection. Some trauma and orthopedic units and some emergency wards have also altered their admission criteria [16, 17, 22]. Despite having suffered an injury, some patients, particularly the elderly and those with comorbidities, have avoided seeking medical help at emergency or trauma and orthopedic wards due to the fear of contracting COVID-19 [13, 22]. In order to increase the safety of the medical personnel and reduce the number of admitted and operated patients, some hospitals increased the number of indications for conservative treatment of injuries and postponing surgery [25, 26].

DRF can be treated surgically with various stabilization methods and non-surgically in plaster [2, 6, 7, 9–11, 25]. The British Orthopedic Association (BOA) recommends non-surgical treatment of DRF during the COVID-19 pandemic. The guidelines accept in some patients the possibility of complications and deformity, that will require deferred surger [25]. The goal of treating injuries during the COVID-19 pandemic is rapid and safe treatment [25].

The reduction of the number of patients treated surgically with DRF during the COVID-19 pandemic may have consequences in the form of more complications in the future [25, 26]. Surgical treatment of DRF is indicated especially in young patients with dislocated, multi-fragmented and intra-articular fractures. In addition to the above factors, the epidemiology of DRF during the COVID-19 pandemic may be influenced by other factors, such as the medical and bioethical framework, the surgeon, and hospital policy (confounding factors) [25, 26]*.*

There have been few studies evaluating the important issue of the impact of the COVID-19 pandemic on DRF epidemiology in children and adults [13, 15, 25].

Nabian et al. presented an epidemiologic model of pediatric injuries during the COVID-19 pandemic based on data from a tertiary trauma center in Iran [13]. Those authors observed an increased proportion of DRFs in children (from 28% of all fractures from the pre-pandemic period to 30% of all fractures during the COVID-19 pandemic [13]. Nabian reported no changes in either the mean age of patients or the male-to-female patient ratio during the COVID-19 pandemic [13]. Bram et al. assessed the effects of the COVID-19 pandemic on the epidemiology of injuries in pediatric patients [15]. According to their report, the total number of fractures decreased by 61%, there were no changes in the male-to-female ratio, and the mean age of patients decreased from 9.4 to 7.5 years [15]. Baawa-Ameyaw reported that 54% of 92 patients with DRF managed nonoperatively during the COVID-19 pandemic had indication for operative management [25].

The sparse available literature on the effect of the COVID-19 pandemic on DRF epidemiology focuses on pediatric patients and has a limited scope, since the authors typically assess the number of patients with specific fracture locations presenting at emergency departments [13, 15].

Nonetheless, there are no detailed data on the possible changes in the epidemiology and treatment of DRFs in children and adults. Such data may prove useful in preparing resources. The research question was whether the COVID-19 pandemic was influencing changes in the epidemiology and treatment of DRFs in children and adults.

The purpose of our study was a comprehensive assessment of the impact of the COVID-19 pandemic on DRF epidemiology, including both children and adults and various fracture fixation methods in two large trauma centers in Poland.

## Methods

Distal radius fracture epidemiology was evaluated in two large trauma centers for pediatric and adult patients in Poland. Outpatients seeking medical attention at emergency departments and inpatients undergoing surgery at trauma-orthopedic wards were evaluated. In order to collect data for the study, the medical database of all data of patients treated in two trauma centers was analyzed. The analysis included the period of the COVID-19 pandemic in Poland (from March 15 to October 15, 2020), and the obtained data were compared with those from the corresponding period prior to the COVID-19 pandemic (from March 15 to October 15, 2019).

The inclusion criteria were a history of DRF in the period between Mar. 15, 2020, and Oct. 15, 2020, or between Mar. 15, 2019, and Oct. 15, 2019; available medical records; and available demographic data. The study was approved by the local review board. All procedures were followed in accordance with relevant guidelines.

Analysis of two databases from two large trauma centers in Poland included the total number of DRF patients, total number of pediatric patients (< 18 years old) with a DRF, total number of adult patients (> 18 years old) with a DRF, total number of pediatric patients with a DRF who received conservative treatment (plaster cast), total number of adults with a DRF who received conservative treatment (plaster cast), total number of pediatric patients with a DRF who received surgical treatment, proportion of pediatric patients who received surgical treatment (total number of all pediatric patients treated surgically/ total number of all pediatric patients × 100%), total number of adults with a DRF who underwent surgical treatment, proportion of adults who underwent surgical treatment (total number of adults who underwent surgical treatment / total number of adults × 100%), total number of adults with a DRF who underwent surgical treatment involving open reduction and volar plate fixation, total number of adults with a DRF who underwent surgical treatment involving closed reduction and Kirschner wire fixation, mean age of all patients, mean age of all adult patients, mean age of all pediatric patients, mean hospital stay duration of surgically treated adults, mean hospital stay duration of surgically treated pediatric patients, and male-to-female patient ratio. All these data for the period of the COVID-19 pandemic in Poland (from Mar. 15 to Oct. 15, 2020) were compared with the corresponding data for the period prior to the COVID-19 pandemic in Poland (from Mar. 15 to Oct. 15, 2019).

The obtained data were statistically analyzed using the Statistica 13.1 program. Pearson’s chi-square test was used to assess the relationship between the frequency distribution of responses in one variable with respect to the other variable. Student’s t-test were used to compare the continuous variables for two groups (during and before the pandemic). The adopted significance level was α = 0.05.

## Results

**Fig. 1:**
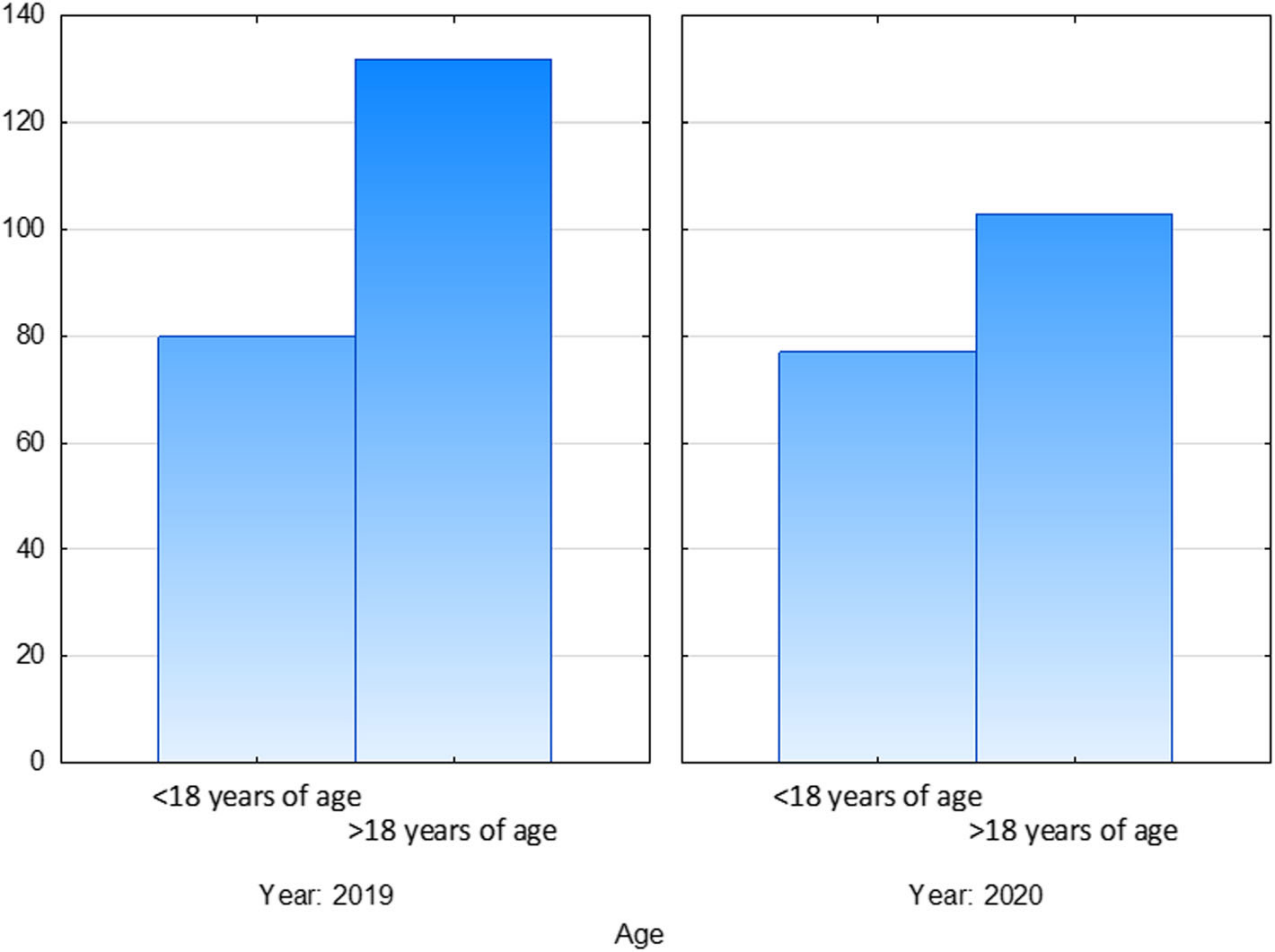
number of adults and children

The results have been presented in Table 1. Our analysis showed that the total number of patients hospitalized due to DRF during the pandemic (in 2020) was 15.1% lower than that from the pre-COVID-19 pandemic period (in 2019). In the case of adult patients, the total number of those hospitalized during the pandemic decreased significantly (by 22%) from 132 to 103 patients, (*p* = 0,01253), Fig. 1. In the case of patients under the age of 18 years, the total number of those hospitalized decreased by 3.8%.

**Table 1:** epidemiological characteristics of patients with distal radius fracture

Variable	2020 (pandemic)	2019 (no pandemic)	the difference between2020 and 2019	P
total number of patients	180	212	−15.10%	0.44816
number of children (< 18 years of age)	77	80	−3.80%	0.31
number of adults (> 18 years of age)	103	132	−22%	0,01253*
the number of children treated conservatively	64	69	−7.20%	0.37679
the number of adults treated conservatively	83	119	−30.30%	0,03618*
number of children treated surgically	13	11	18.20%	0.37679
% of children treated with surgery	16.9	13.8	22.50%	
number of adults treated surgically	20	13	53.80%	0,03618*
% of adults treated with surgery	19.4	9.8	98%	
number of adults treated surgically - volar plate	11	4	275%	
number of adults treated surgically - Kirschner wires	9	9	0%	0.78903
mean age of all patients	37 years, 2 months	40 years, 1 month	−7.20%	0.29329
mean age of adult patients	57 years, 9 months	58 years	−0.20%	0.94019
mean age of childhood patients	9 years, 8 months	10 years, 6 months	−7.50%	0.11946
average length of hospitalization in case of adult surgery [days]	2.55	2.92	−12.70%	0.40053
average length of hospitalization in case of surgical treatmentof children [days]	3.38	3.82	−11.50%	0,03857*
the ratio of men to women	1.5	0.72	208.30%	0.62895
* - p<0.05

**Fig. 2:**
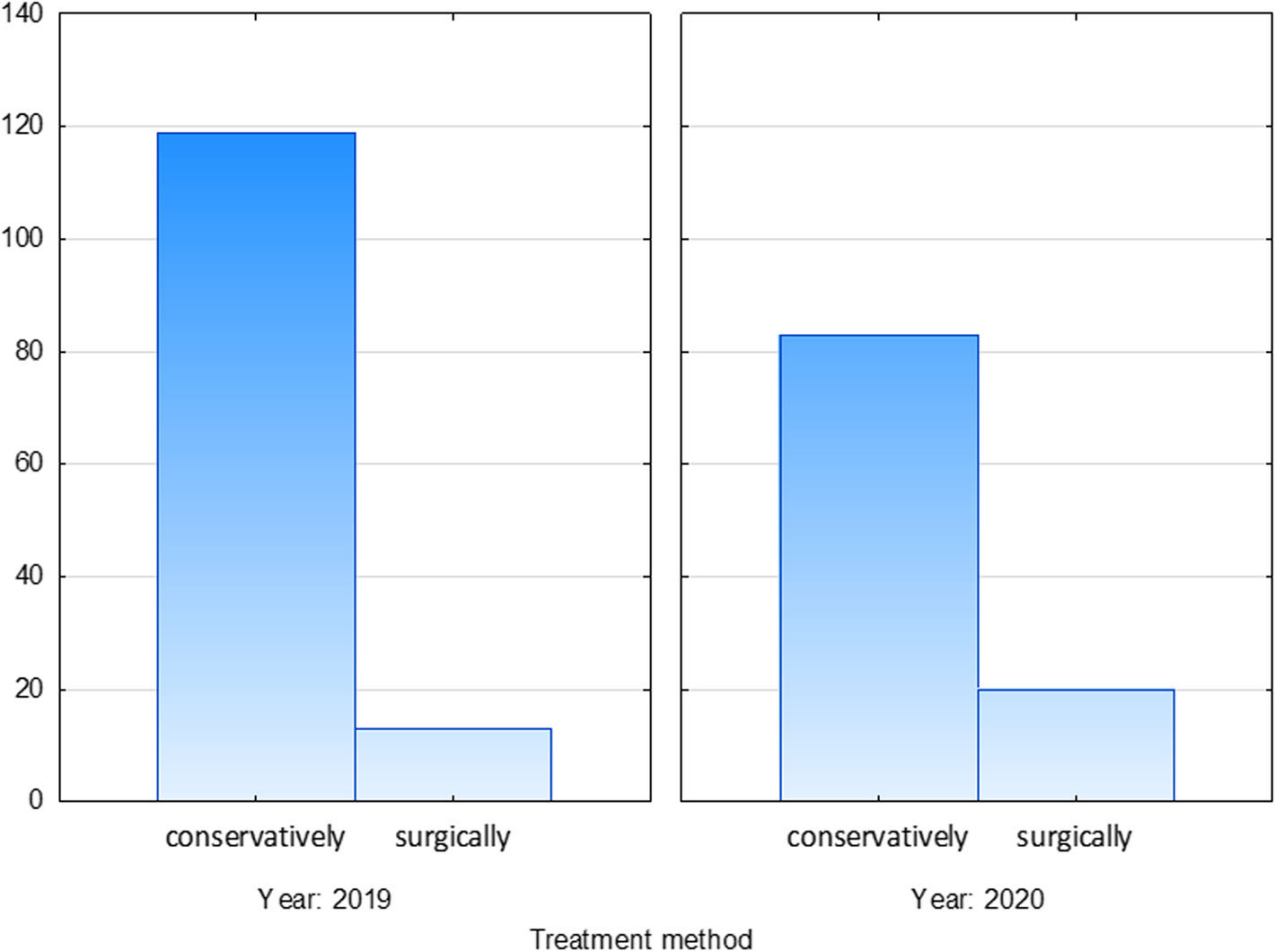
number of surgically and conservatively treated adults

Analysis of the individual treatment methods revealed that the number of adults who underwent conservative treatment was considerably (by 30.3%) significantly lower in the period of the COVID-19 pandemic (*p* = 0,03618), Fig. 2. The number of pediatric patients who underwent conservative treatment decreased somewhat less dramatically (by 7.2%) from the first evaluated period to the second. Compared to the figures from the pre-pandemic period, the number of surgically treated adults was significantly higher (by 53.8%), (*p* = 0,03618), Fig. 2, while the number of surgically treated pediatric patients was higher by 18.2% in 2020.

The parameter that increased the most (by 275%) in comparison to its pre-pandemic value was the number of adults who underwent surgical treatment with volar plate fixation. Interestingly, the number of patients treated with Kirschner wires remained unchanged.

What also draws attention is the lower mean age of patients hospitalized due to a DRF during the pandemic (37 years and 2 months) in comparison to the pre-pandemic mean age of hospitalized DRF patients (40 years and 1 month). In adult patients, the mean age dropped from 58 years to 57 years and 9 months, while in children it dropped from 10 years and 6 months to 9 years and 8 months.

**Fig. 3:**
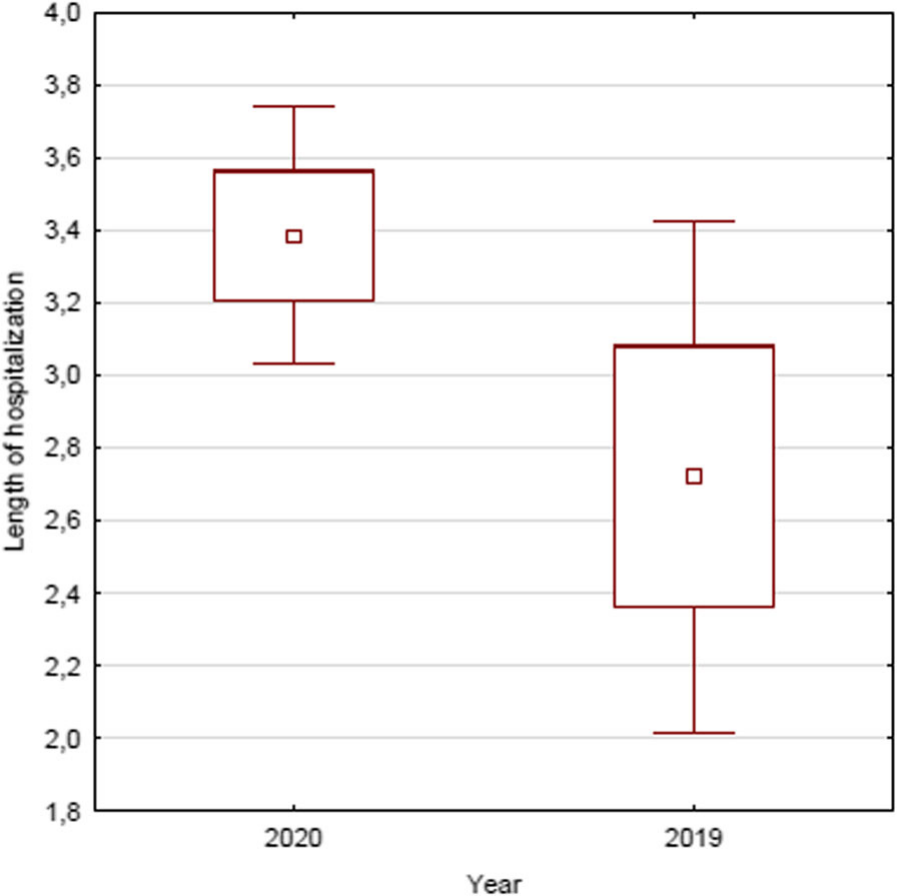
length of hospitalization in case of surgical treatment of children [days]

Our analyses showed hospitalizations of surgically treated adults to be shorter by 12.7% during the pandemic (from 2,92 days in 2019 to 2,55 days in 2020), with the corresponding hospitalizations of surgically treated pediatric patients to be significantly shorter by 11.5% (from 3,82 days in 2019 to 3,38 days in 2020), (*p* = 0,03857), Fig. 3.

The COVID-19 pandemic have shown a considerably increased (by 208.3%) male-to-female ratio among DRF patients (from 0,72 in 2019 to 1,5 in 2020).

## Discussion

Distal radius fractures constitute a social problem [1–13]. The COVID-19 pandemic has had a considerable impact on the lives of both adults and children around the world [13–26]. Some schools have been closed, causing children to stay at home, and some adults work from home. Moreover, during some of the COVID-19 pandemic period, people’s ability to move about freely outdoors and do sports was limited due to government-imposed restrictions. All the above factors affected DRF epidemiology in adults and children.

There have been no studies comprehensively evaluating the important issue of epidemiology and treatment of DRFs in adult and pediatric patients during the COVID-19 pandemic.

Baawa-Ameyaw reported that 54% of 92 patients with DRF managed nonoperatively during the COVID-19 pandemic had indication for operative management [25]. Nabian et al. presented an epidemiologic model of pediatric injuries during the COVID-19 pandemic based on data from a tertiary trauma center in Iran [13]. Those authors observed an increased proportion of DRFs in children (from 28% of all fractures from the pre-pandemic period to 30% of all fractures during the COVID-19 pandemic [13]. Nabian reported no changes in either the mean age of patients or the male-to-female patient ratio during the COVID-19 pandemic [13]. Bram et al. assessed the effects of the COVID-19 pandemic on the epidemiology of injuries in pediatric patients [15]. According to their report, the total number of fractures decreased by 61%, there were no changes in the male-to-female ratio, and the mean age of patients decreased from 9.4 to 7.5 years [15]. Bram et al. noted a decreased incidence of injuries due to sports and other outdoor activities, with an increased incidence of high-energy injuries due to falls from trampolines and bicycles [15]. Hashmi reported a 50% decrease in both elective and emergency admissions to orthopedic wards, with no changes in either the mean age or male-to-female ratio in patients in the COVID-19 pandemic period in comparison with the relevant pre-pandemic figs [16].. Yu et al. observed a 42% decrease in the number of patients with fractures seen at one of the orthopedic wards in China during the COVID-19 epidemic [17]. Poggetti et al. reported a 28.6% decrease in the number of patients undergoing surgery due to hand and wrist trauma in one of Italian hospitals during the COVID-19 pandemic [18]. In one of the Turkish hospitals, the total number of fractures recorded during the COVID-19 pandemic was by 61.6% lower than the number of fractures recorded in 2019 [20].

Our retrospective study showed reduced numbers of pediatric (by 3.8%) and adult patients (by 22%) referred to emergency departments due to DRFs during the COVID-19 pandemic. Similar, or even more pronounced decreases over the COVID-19 pandemic period (compared to period prior to the COVID-19 pandemic) have been reported in other countries (19–69%) [13, 15–22, 24].

The reduced numbers of DRF-associated hospitalizations can be explained by lockdown measures, limited exercise opportunities, and the necessity to stay indoors during the pandemic. As a result of having to stay at home under adult supervision, children and adolescents under the age of 18 years were less prone to suffer injuries, which are typically exercise-related in this age group. Hence the less pronounced difference observed in this age group. Young adults limited their exercise by staying at home; this made them less prone to injuries/falls, which are the most common mechanism of DRFs. The elderly stayed mostly at home due to fears of infection. Some of them did not seek medical attention despite their injury and let it heal without any orthopedic intervention.

We expected to see a trend towards lower numbers of DRF patients due to social distancing measures and instances of self-quarantine, which altered people’s behaviors and lifestyles [13, 18–20, 22–24]. Approximately 25% of injuries in children are due to sports [15]. Sports activities and training sessions were mostly canceled, with schools, kindergartens, and nurseries partly or completely closed. The amount of traffic also declined dramatically due to the COVID-19 pandemic. These factors, as well as the patients’ and their guardians’ fears of infection during a visit to the hospital affected the epidemiology and treatment of DRFs in children and adults [13–17, 23, 24]. Some authors reported falling numbers of traffic accidents, sports-related injuries, and outdoor injuries during the pandemic, which would lead to lower numbers of high-energy fractures [14, 17–19, 22, 24]. However, the number of low-energy fractures remains unchanged [14, 18, 19]. On the other hand, the period of COVID-19 pandemic saw increased numbers of indoor injuries and alcohol-related injuries [14, 17–19, 22, 24].

Evaluating the individual treatment methods, we assumed that most high-energy fractures would require surgical treatment, with most low-energy injuries managed conservatively. Turgut et al. observed an 89% increase in the proportion of children undergoing surgery due to fractures during the COVID-19 pandemic, with no corresponding increase in adults undergoing surgical treatment [20]. Pichard reported an increased proportion of patients undergoing surgery (from 36.9% in 2019 to 51.2% during the COVID-19 pandemic) [24]. We observed increased numbers of patients undergoing orthopedic surgery treatment during the pandemic (an 18.2% increase in the number of children and a 53.8% increase in the number of adults). This may have been a result of the increased numbers of high-energy injuries due to falls from a trampoline or bicycle [15].

The lower by 30.3% number of adult patients receiving conservative treatment can be attributed to limited exercise and recreational activities, whereas the dramatic 98% increase in the proportion of surgically treated adults can be attributed to the work and renovations done around the house during the lockdown period and the maintained high level of activity on the part of construction businesses, which were exempt from lockdown restrictions. This can be best seen while analyzing the number of patients treated with a volar plate. These were mostly patients with high-energy injuries due to falls from a height associated with work done in or around the house and with construction activities.

Our analysis revealed a 7.2% decrease in the mean age of patients during the pandemic, which may have been a result of elderly people’s fears of visiting an emergency department during the pandemic and the more effective measures to prevent injuries in the elderly. On the other hand, Lv et al. reported a significant increase in the mean age of patients presenting with fractures during the pandemic in China [23]. The lower mean age of patients hospitalized due to DRF can be attributed to the nature of the SARS-CoV-2 virus, which is more virulent in the elderly [13]. Because of their fear of infection, elderly patients submitted more eagerly to lockdown restrictions. Moreover, some of the oldest patients never reached a hospital due to fears of infection and allowed their fractures to heal without seeking medical attention.

Our analyses were based on data collected from hospital departments performing elective and emergency procedures. The observed shorter mean hospital stays of patients undergoing surgery during the lockdown period was a result of elective procedures being cancelled, patients with injuries being treated more speedily, and the hospital stays being limited to a minimum due to the epidemiological situation in hospitals. This also applied to pediatric patients who were hospitalized together with an adult guardian.

The increased number of DRFs in males in comparison to that in females can be attributed to uninterrupted work involving physical labor in construction, mining, and smelting industries, despite lockdown restrictions elsewhere.

The increased male-to-female ratio among DRF patients is also associated with the differences in the type of work done by men and women. Jobs requiring physical labor, which tend to be more commonly held by men were exempt from lockdown restrictions, which increased the proportion of men who incurred injuries. Moreover, men who self-quarantined at home remained actively involved in work around the house and in renovations. The women who stayed at home were more likely to engage in low-energy activities, such as cleaning or childcare, which are less traumatic and less likely to cause DRFs.

The limitation of our work may be the fact, that the epidemiology of DRF during the COVID-19 pandemic may be influenced by other factors, such as medical and bioethical framework, the surgeon, and hospital policy (confounding factors) [25, 26]*.*

Our study showed the effect of the COVID-19 pandemic on the epidemiology of DRFs in adults and children.

The general tendency for DRFs to occur decreased during the pandemic; however, the observed increase in the proportion of patients who underwent surgical treatment may be an important warning sign, indicating that the pandemic was responsible for the increased number of high-energy DRFs requiring surgery.

The results of our analysis can be useful in taking appropriate measures and securing the resources necessary for the treatment of DRFs, especially since the COVID-19 pandemic saw increased numbers of DRF patients undergoing surgical treatment.

Moreover, this study suggests the need to inform men about the risk of DRFs, as evidenced by the dramatic increase in the number of male patients with this type of injury.

## Conclusions

Our study showed a significant impact of the COVID-19 pandemic on the epidemiology and treatment of DRFs in children and adults.

We found decreased numbers of pediatric and adult patients with DRFs who were referred to trauma centers during the COVID-19 pandemic.

The COVID-19 pandemic caused an increase in the number of children and significant increase of adults undergoing surgical treatment for DRFs, decreased number of patients in both groups adults and children treated conservative.

We noted an significant increase in the number of adults treated with volar plate, a decrease in mean patient age, significantly shorter durations of hospital stay in children and adults undergoing surgical treatment, and increased number of men treated with DRFs.

## Data Availability

The datasets used and/or analysed during the current study are available from the corresponding author on reasonable request.
